# Gestational Folic Acid Administration Alleviated Maternal Postpartum Emotional and Cognitive Dysfunction in Mice

**DOI:** 10.3389/fphar.2021.701009

**Published:** 2021-06-11

**Authors:** Qianyu Zhang, Qianwen Huang, Li Yao, Wenjuan Liu, Jianxing Ruan, Yingqi Nong, Ye Chen, Lin Fan, Jinyan Wei, Songlu Wang, Li Sun, Hao Li, Yan Zhang, Xiqian Zhang, Fenghua Liu

**Affiliations:** ^1^ Department of Reproductive Health and Infertility, Guangdong Women and Children Hospital, Guangzhou, China; ^2^ Medical Genetics Center, Guangdong Women and Children Hospital, Guangzhou, China

**Keywords:** folic acid, postpartum, behavior, hippocampus, brain-derived neurotrophic factor

## Abstract

Gestational folic acid (FA) supplementation has been widely recognized for its benefits in preventing offspring defects, but its effect on postpartum females has not yet been adequately assessed. The occurrence of emotional and cognitive dysfunction is common in postpartum women, and its treatment remains limited. Considering the promising results of FA in various psychiatric disorders both in human and redents, we tested the effect of gestational FA administration on postpartum psychiatric behavioral phenotypes and the implicated brain-related mechanisms in a murine model. FA was administered orally in both the hormone-stimulated-pregnancy (HSP) model and pregnant mice at doses of 1 and 5 mg/kg. Postpartum behavioral results showed that the disorders of cognitive performance, depressive, and anxiety-related behaviors were all alleviated in the 5 mg/kg FA group. However, the general development of their offspring remained unaffected. Immunofluorescence and immunoblot results revealed that FA pretreatment significantly activated the maternal hippocampal BDNF-related pathway. Morphological studies have confirmed that FA promotes hippocampal neurogenesis. Moreover, synaptic plasticity and synaptic transmission are enhanced. All of these hippocampal changes play critical roles in rescuing neuronal function and behaviors. Thus, our data suggest that gestational FA administration has a therapeutic effect that improves cognition and reduces depression and anxiety in a murine postpartum model. This may be developed as a preventive and adjuvant therapeutic option for pregnant women.

## Introduction

Women who undergo pregnancy and childbirth experience dramatic hormonal fluctuations, which contribute to the occurrence of mental symptoms, including cognitive and emotional dysfunction, as are widely observed in postpartum women. Postpartum mental symptoms include postpartum cognitive dysfunction (PCD), postpartum depression (PPD), and postpartum anxiety (PPA), which have turned into a serious epidemiological concern in the field of maternity care. Pregnant women have significantly poorer cognitive abilities (Henry and Sherwin, 2012). Simultaneously, women are twice as likely to develop depression, with comorbid generalized anxiety disorder; most episodes begin postpartum (Wisner et al., 2013). The incidence and prevalence of unique symptoms occurring after birth range from 8 to 26%, and multiple symptoms are often found concurrently (Fairbrother et al., 2016; Meena et al., 2016; Shorey et al., 2018. However, these symptoms in postpartum women are often overlooked, leading to lower diagnosis rates and even lower treatment rates. These symptoms not only affect the quality of life of postpartum women, but also mother-infant interactions and spousal relationships, which in turn affect child-rearing behaviors and healthy development of infants. The conventional treatment for these symptoms often abides by conventional drug treatment protocols. Even if clinically effective, these still might be rejected by pregnant women considering potential risks to the baby (Noble, 2005). Therefore, therapies that can serve as complementary or alternative treatments must be developed.

The hippocampus plays a central role in the processing of emotional and cognitive behaviors (Korotkova et al., 2018). Unlike general major depression disorder and cognitive impairment, with pathogenic mechanisms associated with psychosocial stress or neurological injury, postpartum symptoms are often speculated to be caused by hormonal fluctuations (Becker et al., 2016; Brown and Schaffir, 2019). A growing number of studies have shown that hippocampus-related mechanisms are implicated in the pathogenesis of postpartum emotional and cognitive dysfunction (Pawluski and Galea, 2007; Baka et al., 2017). Anatomical hippocampal neurogenesis and synaptic plasticity functional dysregulation have been shown to mediate the presentation of certain types of cognitive or affective dysfunction behaviors (Brummelte and Galea, 2016; Workman et al., 2012). Thus, discovery of novel therapies targeting these potential candidates targets is urgent and beneficial for these treatment.

The transition to motherhood not only involves hormones, but also the metabolic adaptations of various nutrients that modify behavioral states. Folic acid (FA), an essential B vitamin, is involved in regulating fetal development during pregnancy, and is required at an even higher dose during pregnancy (Chitayat et al., 2016). A consensus regarding the prevention of neural tube defects and other folic acid-sensitive congenital malformations has been internationally suggested. Moreover, existing clinical studies have shown that FA supplementation during pregnancy is correlated with a lower risk of postpartum mental symptoms (Yan et al., 2017). In fact, FA has shown favorable therapeutic effects in multiple experimental and clinical mental symptom models (Fava and Mischoulon, 2009; Shrestha and Singh, 2013; Budni et al., 2013; Lv et al., 2019). Based on this wide range of therapeutic effects of FA, considering that postpartum mental symptoms almost share the same symptoms as the previously mentioned mental illnesses, characterized by like lower cognitive performance, depression, hopelessness, and anxiety, FA offers promise in terms of its clinical benefits, and may represent a cure of PCD, PPD, and PAD. In this study, we sought to assess this potential.

The etiopathogenesis of emotional and cognitive dysfunction within the postpartum period and has been well established in mice (Zhang et al., 2016). In the first part of this study, a hormone-stimulated pregnancy (HSP) “pseudo-gestational” model was established to mimic the effects of hormones on pregnancy in mice, and the effect of FA administration on related behavioral phenotypes was monitored. In addition, these observations were conducted in naturally pregnant female mice. The effect of the gestational administration of FA on offspring generation was also monitored. This observation may shed light on potential postpartum-related maternal mental symptom prevention and treatment strategies.

## Materials and Methods

### Animals

Ten to twelve-week-old male/female C57BL/6 mice (purchased from Beijing Vitong Lihua Co., Ltd.) were raised in an SPF environment at a temperature of 22–24°C and relative humidity of 55–65%. All animals had free access to food and drinking water and were reared according to a 12/12 light-dark cycle. All experimental animal procedures strictly followed the guidelines of the National Institutes of Health Guide for the Care and Use of Laboratory Animals (NIH Publication No. 85-23, revised 1996) and were authorized by the Ethics Committee of Guangdong Women and Children Hospital. All efforts were made to minimize animal suffering. After the animals were accommodated to environmental condition, then the experimental procedures started on 15–16-weeks-old animals. In behaviral tests, the animal behavior tests were monitored using Ethovision 7.0 software (Noldus, Wageningen, Netherlands).

### Modeling and Folic Acid Administration

In this study, we utilized two postpartum models: the hormone-induced pseudo-pregnancy (HSP) model and the natural delivery postpartum model. The HSP model referred to the modeling method adopted in mice (Zhang et al., 2016; Zhu and Tang, 2020), and animals with bilateral oophorectomy (OVX) were treated with hormones. Briefly, oophorectomy was performed under isoflurane anesthesia in female mice, whereas the control group (Con) underwent a sham operation, except for the oophorectomy. Estrogen (E2) and progesterone (P4) were dissolved in corn oil and subcutaneously injected into OVX mice 9 days after recovery. E2 was administered for 22 consecutive days (20 μg/kg for the first 15 days and 400 μg/kg for the subsequent 7 days), whereas a P4 (32 mg/kg) injection was administered in the first 15 days. Mice in the Con group were injected with a corn oil solvent. Relevant tests were performed within 7 days of E2 withdrawal. In the model of natural pregnancy, mice were caged at a male:female ratio of 1:2, and the appearance of vaginal suppositories was considered the first day of gestation (G0). Pregnant mice and their offspring from postnatal day (PD) were carefully handled and recorded. The body weights of the pregnant mice in each group were measured at G7, G15, and G18. The gestational length, litter size and sex ratio of the offspring, and litter weights during development were recorded until P21. Folic acid was purchased from Sigma Chemical Co. (St. Louis, MO, United States) and dissolved in distilled water. In this study, two doses of folic acid (1 and 5 mg/kg, gavage) were selected as supplements in addition to normal food sources. In the HSP model, folic acid supplementation was administered daily, along with E2, until E2 was discontinued. In the model of natural pregnancy, folic acid supplementation began with the onset of vaginal suppositories until birth.

### Morris Water Maze Test

The MWM was adopted from a previous study (Sun et al., 2020), and the diameter of the water maze device was 1.5 m. It was filled with an opaque water solution dyed with white dye, and the water temperature was maintained at 24°C. The camera device was set above the recording device. The circular area in the device was evenly divided into four quadrants, and the sign markers were set at the periphery of each quadrant. A movable platform was placed 1 cm underwater at a specific position in each quadrant, and each animal tested corresponded to a specific target quadrant. The experiment lasted for 6 days. In the first 5 days, mice were placed in water facing the wall of the pool in one of the four quadrants and the time required to climb up the platform within 90 s was assessed. On day 6, the platform was removed and the total time of the mice passing through the target quadrant within 90 s was recorded. The time ratio was thereafter calculated.

### Spontaneous Alternation Y-Maze Test

The Y-maze is composed of three arms of equal length, randomly defined as the starting arm, new arm, and other arms. The mice were placed in a random arm, named the starting arm, and moved freely to explore the other arms for 5 min. A correct alternation was recorded when the mice entered the three different arms. The spontaneous alternation rate (%) was calculated as the total number of correct alternations/(total crossing times of 3 arms − 2) × 100%.

### Novel Object Recognition Test

The NOR was adopted as previously described (Muha et al., 2019). Briefly, animals were placed in an organic plastic experimental facility (40 cm × 40 cm × 40 cm) for adaptation every day, and then returned to the cage after 10 min of free exploration for three days. On the fourth day, two identical objects (block-shaped toys, Lego Company) were placed in a relative position on one side of the field. The mice were gently placed in the positions of the two objects with their backs to them (familiarization phase). After 10 min of exploration, the animals were removed and returned to the cage. Exploration was defined as directing the nose to the object (distance of <2 cm) and/or touching the object with the nose. The animals showed a preference for objects during familiarization were excluded from the analysis. After 1.5 h, one of the two identical objects was replaced with a different object, named the old object and the new object, respectively. The time the mice spent exploring the two objects was recorded. The NOR index (%) was calculated as (time to explore novel object/total time to explore two objects: time to explore old object/total time to explore two objects) × 100%.

### Open-Field Test

The box for the open field experiment was a square box with the following dimensions: length × width × height = 50 cm × 50 cm × 40 cm. Each mouse was placed in the center of the box, and the activity of the mice was video recorded within 5 min. The residence time in the central region of the square was recorded.

### Forced Swim Test

The forced swim test is a desperate behavior test used to assess depression-like behaviors. The experiment was performed as previously described (Guo et al., 2016). Mice were placed in a transparent plexiglass cylinder (40 cm in height and 20 cm in diameter) filled with water at a depth of 25 cm for 6 min, and the time of immobilization (just for the movement of the head on the water surface) for the last 4 min was recorded.

### Elevated Plus Maze Test

As previously described (Šutulović et al., 2021), the elevated plus maze consists of two open arms and two closed arms. The mice were placed in the test room for 2 h in advance to adapt to allow for their adaptation to the experimental environment. The mice were gently placed in the central position facing the open arm, and the time and number of mice entering the open arm and the total activity trajectory were recorded within a period of 5 min.

### Immunofluorescence

BrdU staining was performed by continuous intraperitoneal injection of BrdU (50 mg/kg) twice daily for 5 days before behavioral tests in advance. After the animals were deeply anesthetized and perfused with ice-cold 0.1% MPBS and 4% paraformaldehyde, the brain tissue was collected and dehydrated in a sucrose solution. Continuous coronal slices were cut into 35 μm slices using a frozen slicer. Hippocampal sections were incubated with 10% BSA (containing 0.3% Triton X-100) at room temperature for 1 h. The corresponding primary antibodies were added and incubated with the sections overnight at 4°C: mouse anti-BDNF (1:500, Abcam; ab203573), rat anti-BrdU (1:100, AbD Serotec; OBT0030), and mouse anti-Nestin (1:1000, Millipore, MAB5326). After washing with PBS, fluorescent secondary antibody (Invitrogen) was added and incubated at room temperature for 2 h without light. After washing with PBS, a DAPI staining solution was added, and the plates were sealed and observed under a confocal microscope (LSM510, Carl Zeiss, Goettingen, Germany). A total of 7–8 sections were randomly selected from each mouse for image acquisition and statistical analysis.

### Immunoblotting Analysis

The animals were sacrificed with CO2 and operated on ice to rapidly isolate the hippocampus. A BCA protein assay kit was used to determine the total protein concentration in the tissue samples. Loading protein was separated by 12% sodium dodecyl sulfate (SDS) -polyacrylamide gel (PAGE) and then transferred to polyvinylidene fluoride membranes (Millipore, Billerica, Massachusetts, United States). After blocking with 5% skimmed milk for 2 h at room temperature, the membranes were incubated overnight at 4°C with primary antibody (anti-BDNF: 1:500, Santa Cruz Biotechnology, United States; anti-TrkB: 1:400, Santa Cruz Biotechnology, United States; anti-p-PIK3: 1:500, Cell Signaling, United States; anti-PIK3: 1:400, Cell Signaling, United States; anti-p-AKT: 1:200, Cell Signaling, United States; anti-AKT: 1:200, Cell Signaling, United States; anti-p-mTOR: 1:400, Cell Signaling, United States; anti-mTOR: 1:400, Cell Signaling, United States; anti-synaptophysin: 1:600, Abcam, United States; anti-Synapsin-1: 1:800, Abcam, United States; anti-PSD95: 1:800, Cell Signaling, United States; anti-GAPDH: 1:600, Santa Cruz Biotechnology, United States). After washing with PBST solution, the membranes were incubated with HRP-conjugated secondary antibody (1:10,000) for 2 h at 25°C. After treatment with ECL, bands were visualized with SuperSignal West Pico Chemiluminescent Substrate (Thermo Fisher Scientific) and analyzed with ImageJ software according to the gray levels of the target protein and reference protein (GAPDH).

### Long-Term Potentiation Electrophysiological Recordings

Mice were anesthetized with sevoflurane and immediately decapitated (Bieschke et al., 2011). Their brain tissue was placed in ACSF (206 glucose, 2.4 KCl, 2.0 MgSO4, 1.0 NaH2PO4, 1.0 CaCl2,25.0 NaHCO3, 1.0 MgCl2, 25 NaHCO3, 10 mM D-glucose, in mM) containing 95% 02 and 5% CO2 at 4°C for 1 min. Subsequently, coronal hippocampal slices of 400 µm thickness were transferred to the above ACSF solution and incubated for 1 h at 37°C. When recording, the temperature in the irrigation groove was kept at 25°C, and the continuous rate of ACSF (pH = 7.4) perfusion flow was set to 1.0–1.5 ml/min. The stimulation electrode uses a bipolar tungsten stimulation electrode, placed on the Schaffer collaterals of CA3 region, to provide a constant current pulse through the stimulator (Sen. 3301, Nihon Kohden, Japan). The recording pipettes were pulled with a standard borosilicate glass tube filled with 2M NaCl, and the impedance was 3-9MQ and were placed on in the stratum radiatum of the CA1 area. The 50% stimulation intensity of the maximum responses of field excitatory postsynaptic potentials (fEPSP) was selected as the final stimulus intensity, and fEPSP over 20 min was recorded as a stable baseline. Data were normalized with respect to the mean values of fEPSP slope recorded during this period. Two consecutive trains (1 s) of stimuli at 100 Hz separated by 20 s were applied to the slices to induce LTP. The recorded electrical signals were amplified with an amplifier (MEZ-8201), and the fEPSP signals were digitized and saved using a pCLAMP system (Axon Instrument Inc.).

### Statistical Analysis

The collected data are shown as the mean ± standard error of the mean (SEM) and analyzed with GraphPad Prism software (version 6.0; GraphPad Inc., La Jolla, CA, United States). Statistical differences between group means were evaluated using a Student’s t-test, one-way analysis of variance (ANOVA) followed by post hoc test, and two-way RM ANOVA according to the test. Statistical significance was set to *p* < 0.05.

## Results

### Hormone-Simulated Pregnancy Postpartum Model Show Impaired Emotional and Cognitive Dysfunction

**FIGURE 1:**
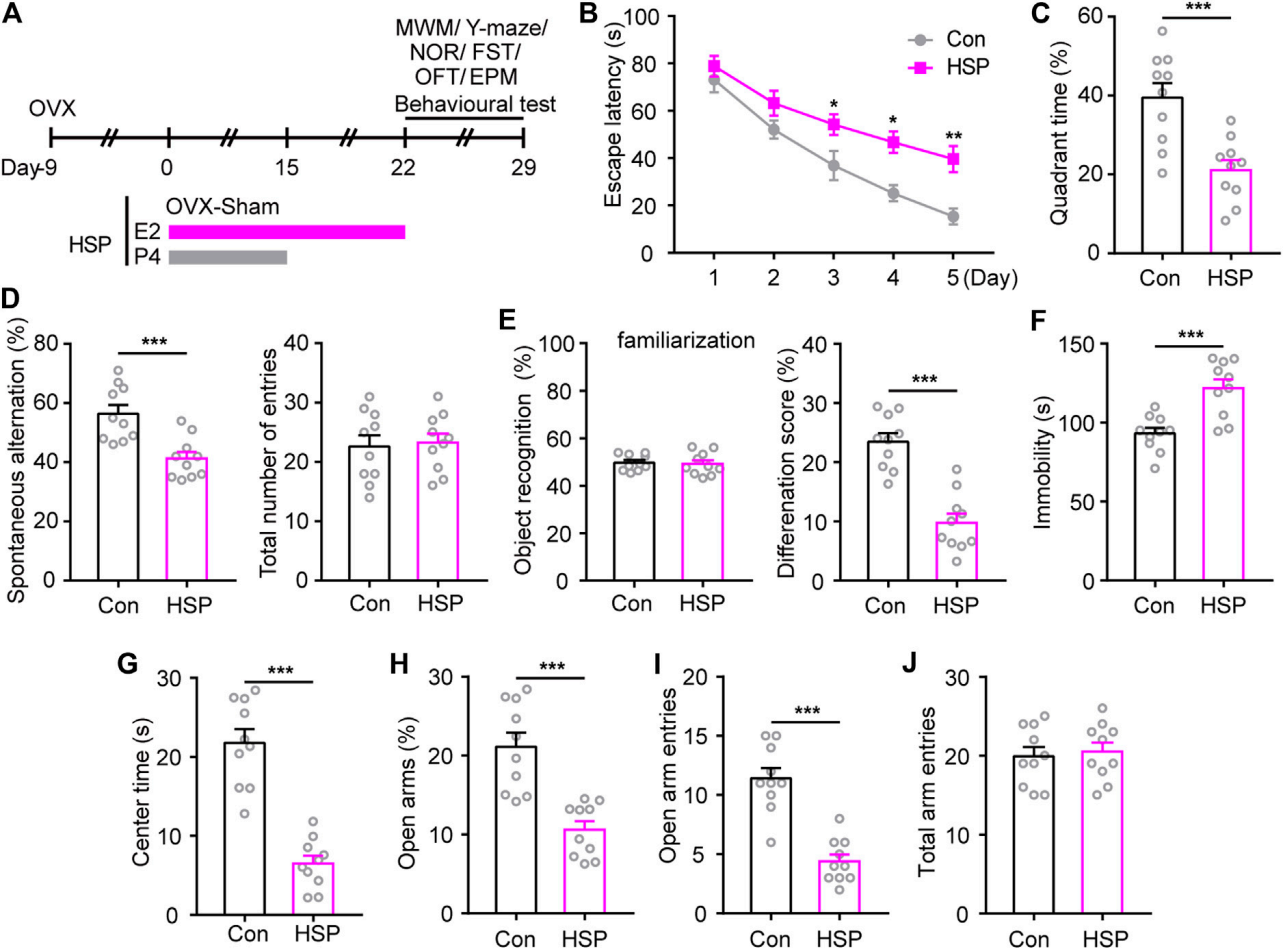
Behavioral changes induced by HSP modeling. **(A)** Schematic timeline representation of HSP modeling and experimental design. **(B)** The latency to reach the hidden platform during the acquisition phase from training days 1–5. The latency during the navigation training was analyzed using two-way repeated measures ANOVA: F (4, 36) = 35.86, *p* < 0.0001. **(C)** The time spent exploring the target quadrant of each group on the 6th day of probe tests with the removed platform. Student’s *t*-test: t = 4.096, *p* = 0.0007. **(D)** The performance of spontaneous alterations and total number of entries in the Y-maze test. Student’s *t*-test: t = 4.118, *p* = 0.0006; Student’s *t*-test: t = 0.2468, *p* = 0.8079. **(E)** The preference for objects during familiarization and performance to distinguish familiar/novel objects in the NOR test. Student’s *t*-test: t = 0.2549, *p* = 0.8017; Student’s *t*-test: t = 6.449, *p* < 0.0001. **(F)** Immobility time in FST. Student’s *t*-test: t = 4.288, *p* = 0.0004. **(G)** Time spent in the central area of the open-field test. Student’s *t*-test: t = 7.649, *p* < 0.0001. Percentage of time spent in the open arm **(H)**, entries into open arms **(I)**, and total arm entries **(J)** in the elevated plus maze (EPM) test. Student’s *t*-test: t = 7.649, *p* < 0.0001; Student’s *t*-test: t = 6.614, *p* < 0.0001; Student’s *t*-test: t = 0.3619, *p* = 0.7217. Data are presented as scatter points plus the mean ± SEM. *n* = 10 for each group. *p < 0.05; **p < 0.01; ***p < 0.001 compared with Con.

An OVX combined with hormone-simulated pregnancy mouse model was utilized to assess potential postpartum cognitive and emotional outcomes, and the behavioral test was begun 7 days after the withdrawal of progesterone (P4) (Figure 1A). The MWM test showed that HSP mice exhibited longer latency to reach the hidden platform during the acquisition phase, and a lower percentage of time to explore the target quadrant during the probe test phase when the platform was removed (Figures 1B,C). Consistently, impaired cognition was also observed in the Y-maze test and novel object recognition memory in HSP female mice compared with the Sham-treated OVX control, while the total number of entries and object recognition during familiarization phase had little changes. (Figures 1D,E). These results indicate that the HSP model induces impaired spatial working memory and short-term non-spatial memory. To assess the effects of HSP on emotion-related behaviors induced by HSP treatment, we performed a FST, OFT, and EPM. The HSP mice showed increased immobility time in the FST (Figure 1F). The open field test and elevated plus maze test were performed to assess patterns of anxiety-like behavior. The analysis of OFT demonstrated that the HSP mice spent significantly less time in the central area within the arena (Figure 1G). In the EPM test, the trajectory diagram showed that the time in the open arms and the number of entries were markedly reduced in the HSP group compared to the control group (Figures 1H,I). No differences were observed between the two groups in the total arm entries in the EPM test (Figure 1J). These results indicate that the HSP model exhibited impaired emotional and cognitive dysfunction.

### Behavioral Analysis for Hormone-Stimulated Pregnancy Model After Folic Acid Treatment

**FIGURE 2:**
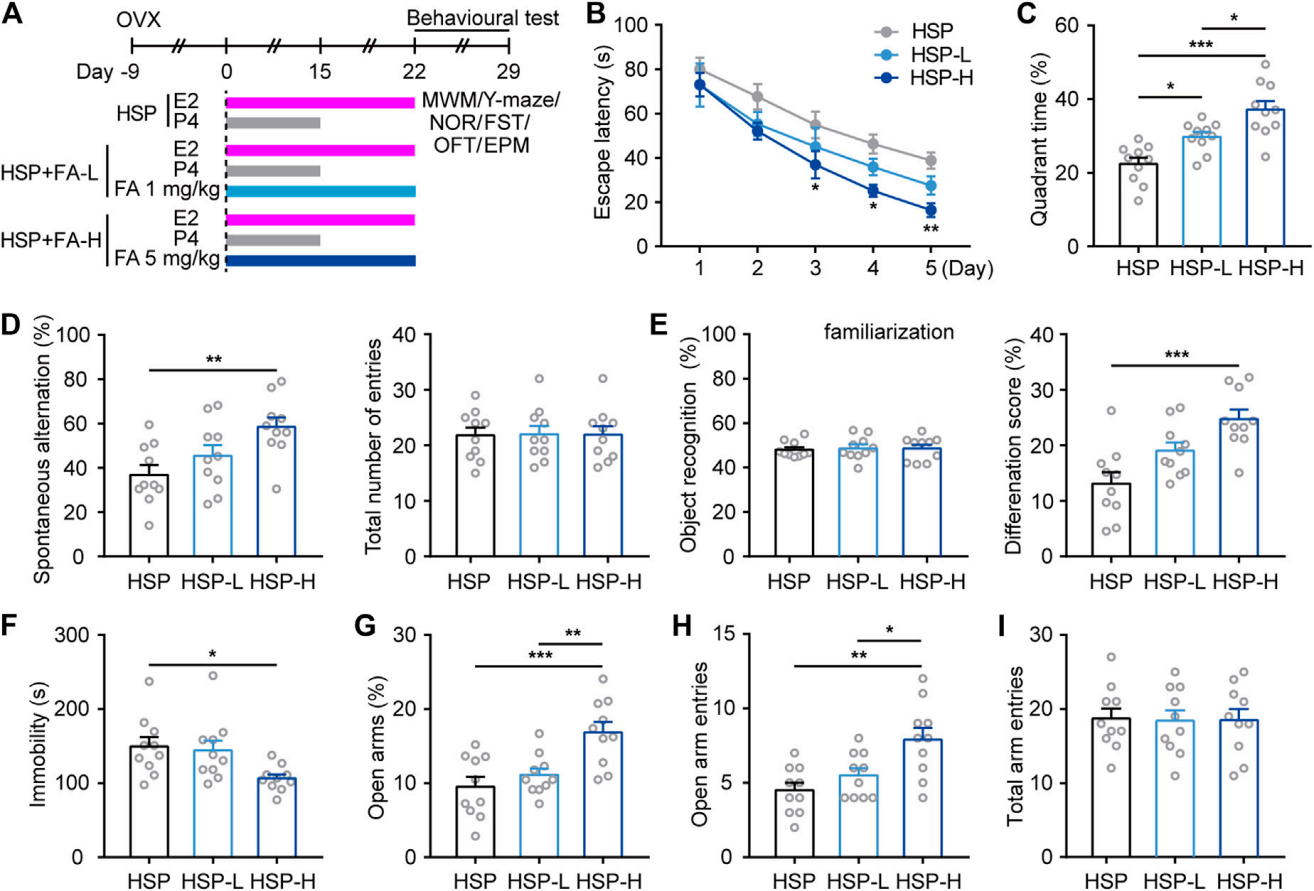
Effects of FA on cognitive- and emotional-related behaviors in female HSP mice. **(A)** Schematic representation of the experimental procedure for assessing the effects of FA administration in HSP mice. **(B)** Escape latency to the hidden platform in the training phase test of the MWM. Two-way repeated measures ANOVA was used to analyze the latency during the navigation training: F (4, 36) = 45.19, *p* < 0.0001. *p < 0.05; **p < 0.01; ***p < 0.001 HSP-H compared with HSP. **(C)** The percentage of time spent exploring the target quadrant. One-way ANOVA: F (2, 27) = 16.31, *p* < 0.0001. **(D)** The spontaneous alteration rate and total number of entries were analyzed in the Y-maze task. One-way ANOVA: F (2, 27) = 5.796, *p* = 0.0081. One-way ANOVA: F (2, 27) = 0.004526, *p* = 0.9955. **(E)** FA significantly improved the ability to discriminate a novel object with a familiar object in HSP mice while had little effect on the preference for objects during familiarization. One-way ANOVA: F (2, 27) = 0.06243, *p* = 0.9396. One-way ANOVA: F (2, 27) = 10.89, *p* = 0.0003. **(F)** FA-treated mice also exert less immobility in the FST. One-way ANOVA: F (2, 27) = 4.464, *p* = 0.0211. **(G)** FA administration increased open arm exploring time in HSP mice. One-way ANOVA: F (2, 27) = 9.777, *p* = 0.0006. FA administration increased open arm entries number in HSP mice **(H)**, with little changes on total arm entries **(I)**. One-way ANOVA: F (2, 27) = F (2, 27) = 15.25, *p* < 0.0001. One-way ANOVA: F (2, 27) = 0.1126, *p* = 0.8939. Data were presented as scatter points plus mean ± SEM. *n* = 10 for each group. *p < 0.05; **p < 0.01; ***p < 0.001.

We then investigated whether cognitive and emotional behavioral impairment in HSP mice could be improved by gestational FA administration. In addition to normal food sources for control HSP mice, considering the dose of normal daily requirement, two supplementary concentrations of FA were chosen to determine whether a therapeutic dose response existed. The HSP mice were orally administered once per day with either FA (1 mg/kg [HSP-L] or 5 mg/kg [HSP-H]) or vehicle (HSP) (Figure 2A) during HSP modeling. Behavioral results showed that animals from the FA-administered groups learned better than the HSP controls, as evidenced by significantly faster escape latencies during training sessions and a longer time spent exploring the platform in the target quadrant during the probe trial of the MWM task (Figures 2B,C). In addition, mice treated with FA were more likely to explore alternate arms in the Y-maze test and spent more time exploring the novel object than the familiar object, compared with the HSP control, while the total number of entries and object recognition during familiarization phase had little changes among three groups (Figures 2D,E). FA-treated HSP mice also showed decreased immobility in the FST (Figure 2F) and displayed increased time and number of entries into the open arms of the EPM as compared to controls (Figures 2G,H), and no differences were observed between the three groups in the total arm entries in the EPM test (Figure 2I). The effect of FA observed in the above results showed that FA had a dose-dependent relationship with a certain behavioral phenotype. A higher dose of FA resulted in a better effect than the low-dose group.

### Effects of Gestational Folic Acid Administration on Postpartum Behavioral Analysis

**FIGURE 3:**
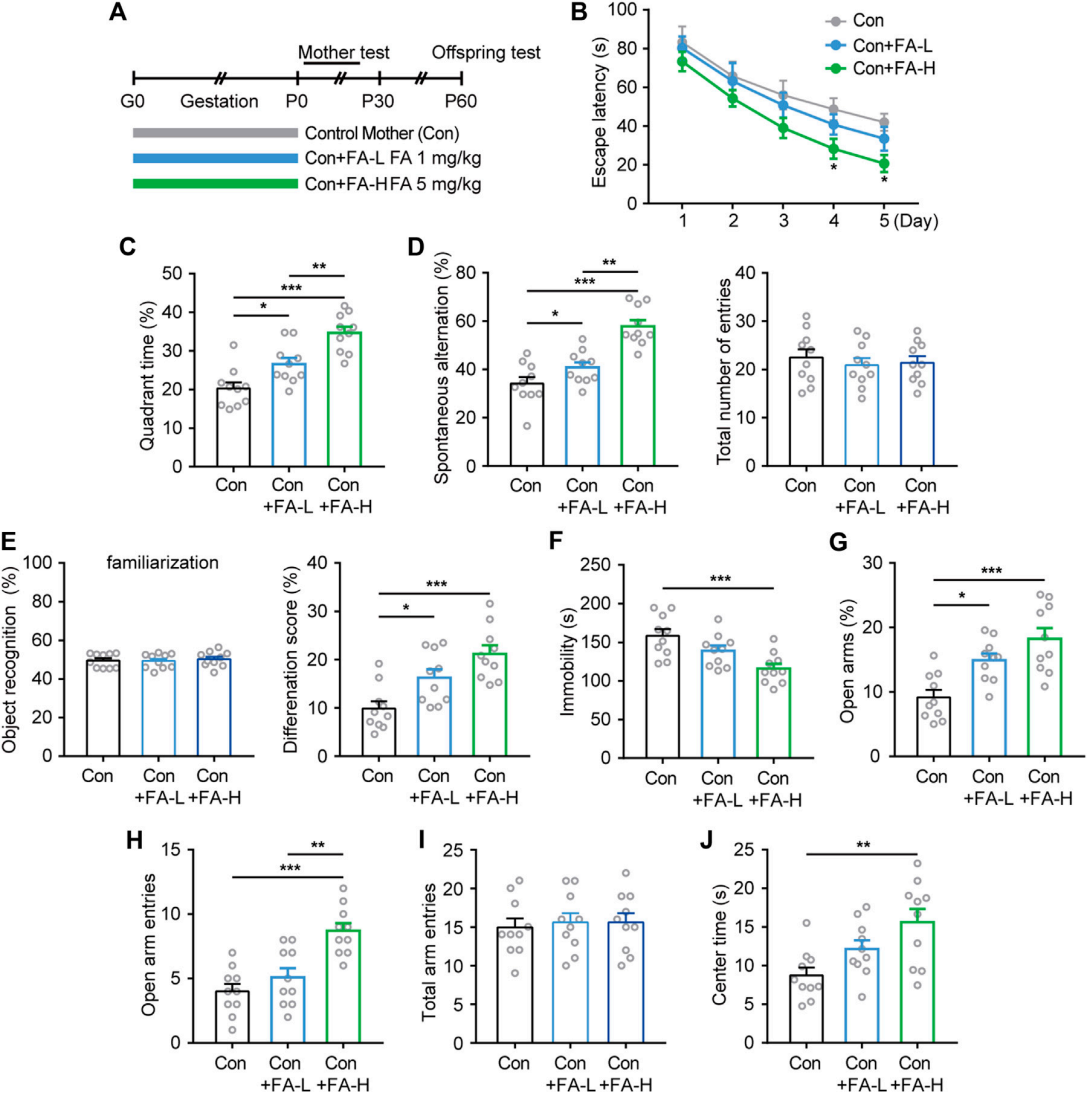
Effects of FA on cognitive- and emotional-related behaviors on postpartum female mice. **(A)** Schematic diagram of the experimental workflow on maternal and offspring. **(B)** Latency to reach the hidden platform in the training phase of the MWM. Two-way repeated measures ANOVA was used to analyze the latency during the navigation training: F (4, 36) = 31.08, *p* < 0.0001. *p < 0.05 Con + FA-H compared with Con. **(C)** FA administration increase the time spent exploring the target quadrant. One-way ANOVA: F (2, 27) = 20.45, *p* < 0.0001. **(D)** The spontaneous alteration rate and total number of entries were analyzed in the Y-maze task. One-way ANOVA: F (2, 27) = 24.46, *p* < 0.0001. One-way ANOVA: F (2, 27) = 0.2525, *p* = 0.7787. **(E)** Novel object discrimination were significantly improved after FA administration in postpartum female mice while had little effect on the preference for objects during familiarization. One-way ANOVA: F (2, 27) = 0.2078, *p* = 0.8137. One-way ANOVA: F (2, 27) = 11.84, *p* = 0.0002. **(F)** FA administration decreased the immobility time in the FST in postpartum female mice. One-way ANOVA: F (2, 27) = 8.665, *p* = 0.0012. The EPM results of time spent in open arms **(G)**, the frequency of entries into open arms **(H)**, and total arm entries **(I)** revealed treatment effects on anxiety. One-way ANOVA: F (2, 27) = 12.36, *p* = 0.0002. One-way ANOVA: F (2, 27) = 15.25, *p* < 0.0001. One-way ANOVA: F (2, 27) = 0.1126, *p* = 0.8939. **(J)** FA administration increased OFT center area exploring in postpartum female mice. One-way ANOVA: F (2, 27) = 6.74, *p* = 0.0042. Data were presented as scatter points plus mean ± SEM. *n* = 10 for each group. *p < 0.05; **p < 0.01; ***p < 0.001.

Next, we investigated whether gestational FA administration could protect against impaired cognitive and emotional performance in natural postpartum female mice and its effect on maternal and offspring characteristics (Figure 3A). Interestingly, the MWM results showed that the escape latency improved more obviously in the FA-H group during both the trial training (Figure 3B) and probe test (Figure 3C). Similarly, FA administration also improved cognitive performance in both the Y-maze and NOR tasks, while the FA administration had little effect on the total number of entries and object recognition during familiarization phase among three groups (Figures 3D,E). Moreover, in the despair-like behavior measured in the FST, the immobility time was significantly shorter in the FA-H group than in the control group (Figure 3F). In the EPM test, the FA-H group mice exhibited more time and entries into the open arms than the control group (Figures 3G,H). No differences were observed between the three groups in the total arm entries in the EPM test (Figure 3I). The OFT study (Figure 3J) further confirmed this trend, suggesting that the FA-H group had a significant improvement in learning, memory, and emotional performance.

### Gestational Folic Acid Administration on Pregnancy Outcomes and Offspring Development

**FIGURE 4:**
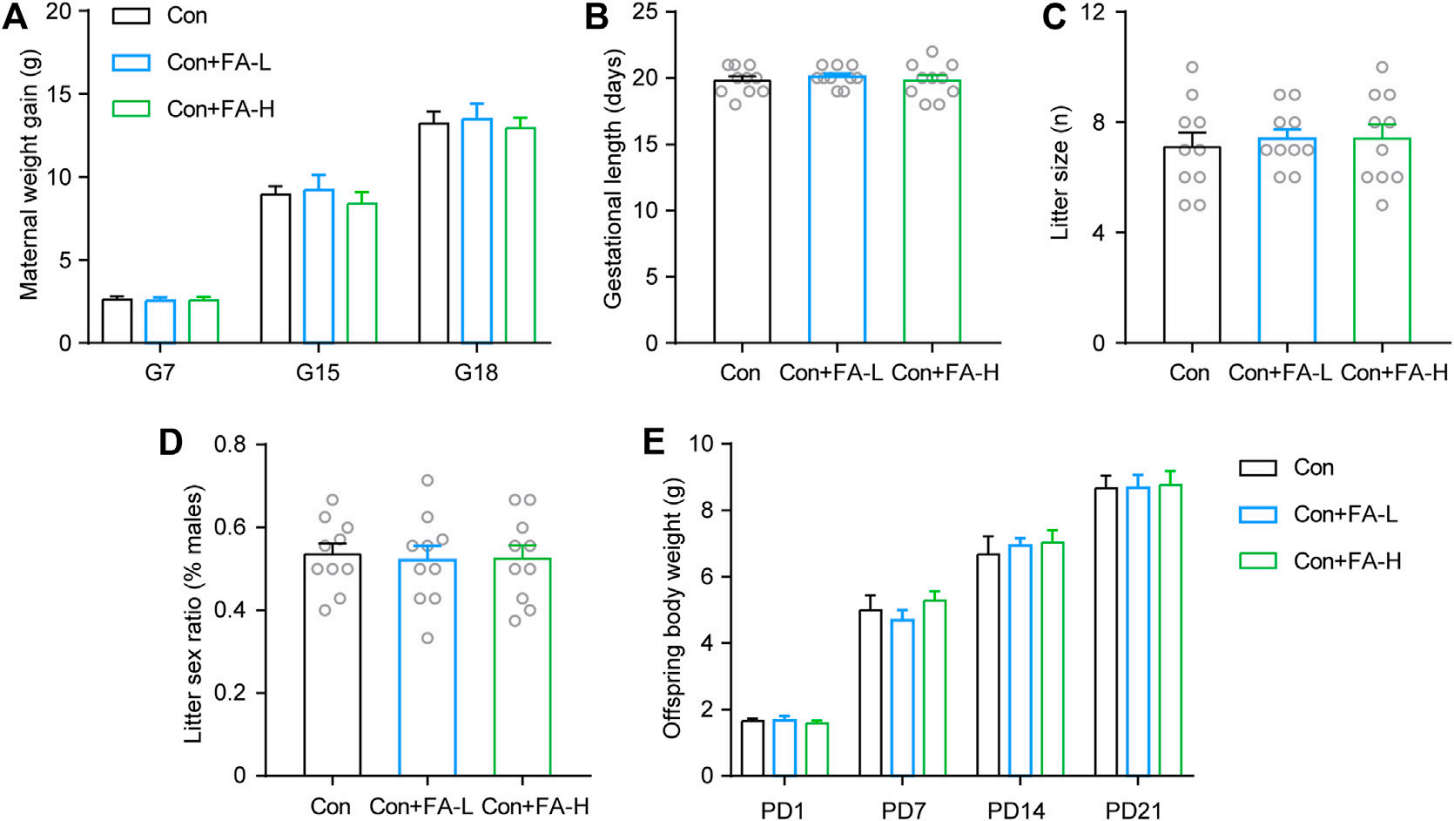
Effects of gestational FA on pregnancy outcomes and offspring characteristics. FA administration during pregnancy had no significant effect on maternal body weight **(A)**, length of gestation **(B)**, dam litter size **(C)**, fraction of pup sex **(D)**, and offspring body weight during development **(E)**. Two-way repeated measures ANOVA was used to analyze the maternal body weight: F (2, 18) = 0.2421, *p* = 0.7875. Length of gestation via One-way ANOVA: F (2, 27) = 0.2691, *p* = 0.7661. Dam litter size via One-way ANOVA: F (2, 27) = 0.1357, *p* = 0.8737. Fraction of pup sex via One-way ANOVA: F (2, 27) = 0.04937, *p* = 0.9519. Two-way repeated measures ANOVA was used to analyze the offspring body weight: F (2, 18) = 0.3204, *p* = 0.7299. Data are presented as scatter points plus the mean ± SEM. *n* = 10 for each group.

Previous FA results on postpartum outcomes were inspiring. Thereafter, we examined whether gestational FA administration had a negative effect on pregnancy or offspring outcomes. FA administration had little effect on maternal body weight gain (Figure 4A), gestational length (Figure 4B), or abortion during pregnancy. No significant differences were observed in the litter size and sex ratio of the offspring (Figures 4C,D). Average pup weights per litter were recorded until P21, and there was no statistical significance in any group (Figure 4E). Gestational FA supplementation had little negative effect on pregnancy and offspring general development.

### Gestational Folic Acid Administration Promotes Hippocampal Neurogenesis in Postpartum Female Mice

**FIGURE 5:**
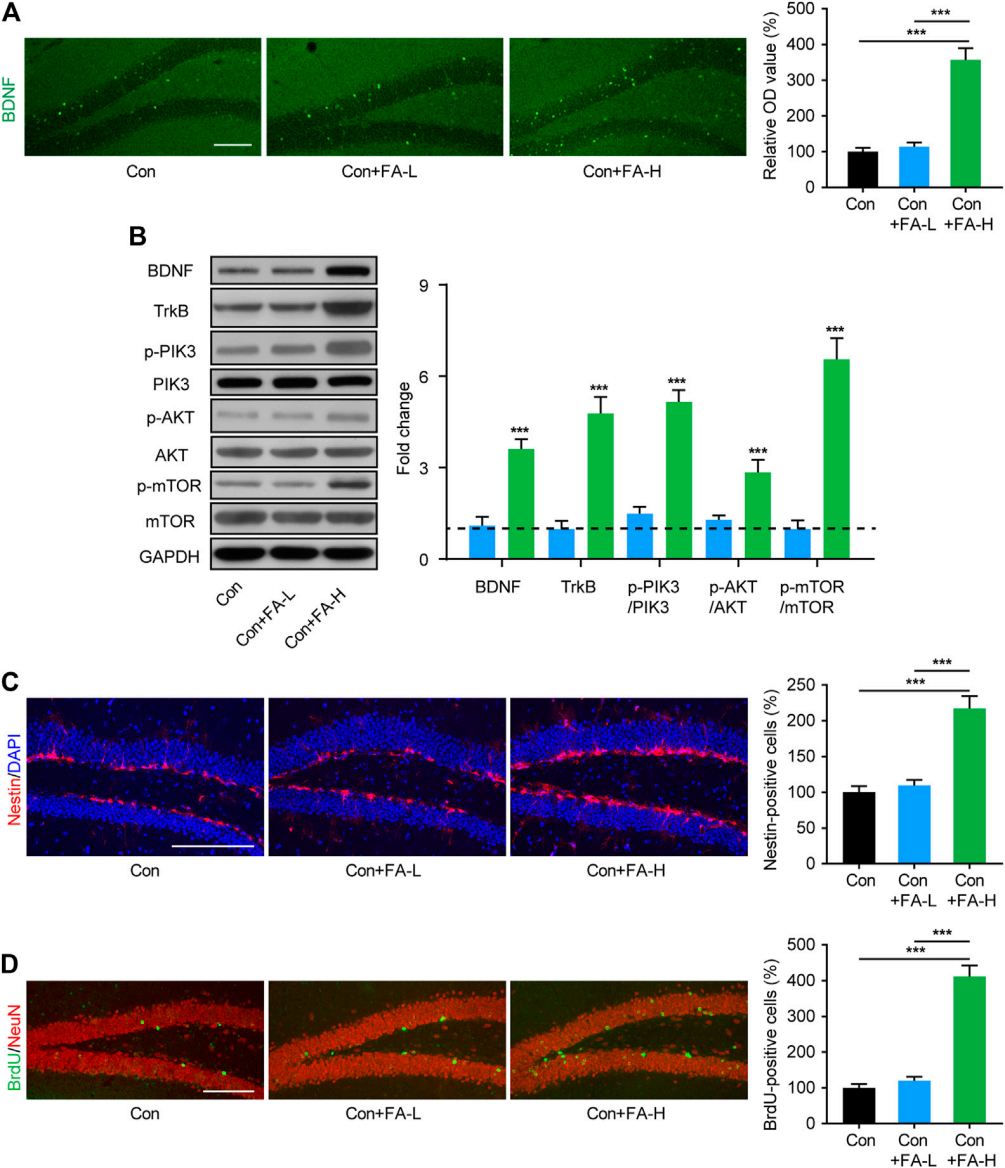
Gestational FA administration promotion of neurogenesis and related molecular expression in postpartum female mice. **(A)** Representative immunohistochemical images for BDNF (Green) in the hippocampus. Histogram showing average fluorescence intensity in O.D. value. One-way ANOVA: F (2, 15) = 47.97, *p* < 0.0001. Scale bar = 100 μm. **(B)** Representative immunoblots and quantifications showing BDNF, TrkB, p-PIK3/PIK3, p-AKT/AKT, and p-mTOR/mTOR expression in the hippocampus. BDNF One-way ANOVA: F (2, 15) = 36.87, *p* < 0.0001. TrkB One-way ANOVA: F (2, 15) = 61.06, *p* < 0.0001. p-PIK3/PIK3 One-way ANOVA: F (2, 15) = 103.2, *p* < 0.0001. p-AKT/AKT One-way ANOVA: F (2, 15) = 21.66, *p* < 0.0001. p-mTOR/mTOR One-way ANOVA: F (2, 15) = 70.2, *p* < 0.0001. The Con group was normalized as a dotted line, and the statistical results were compared with the Con group. **(C)** The immunohistochemistry staining and quantification of Nestin + cells in hippocampus of each group. Nestin (red) and DAPI (blue). One-way ANOVA: F (2, 15) = 28.99, *p* < 0.0001. Scale bar = 100 μm. **(D)** The immunohistochemistry staining and quantification of BrdU + cells in hippocampus of each group. BrdU (green) and NeuN (red). One-way ANOVA: F (2, 15) = 76.08, *p* < 0.0001. Scale bar = 100 μm. Data were presented as mean ± SEM. Scale bar = 100 μm n = 6 for each group. **p < 0.01; ***p < 0.001.

The hippocampus is a key brain region implicated in cognitive and mood disorders. Since neurogenesis plays an important role in the above process and thus contributes to the pathological process of the disease, we assessed neurogenesis-related molecular changes in FA-treated postpartum female mice. Immunofluorescence staining showed increased BDNF expression in the FA-H group mice (Figure 5A). Subsequently, WB assays were conducted to confirm this result. The expression of BDNF in hippocampal tissue was significantly increased in the FA-H group compared to that in the control group (Figure 5B). Further verification was provided by Western blotting analysis of neurogenesis-related pathway proteins. Compared with the control group, in the hippocampus of FA-H group mice, the protein expression of TrkB, p-PIK3, p-AKT, and p-mTOR increased. However, this increase was not associated with an increase in total PIK3, AKT, and mTOR expression (Figure 5B). Hippocampal neurogenesis was evaluated using neuroepithelial stem cells (marked by Nestin) and mitotic process neural stem cells (BrdU) in the dentate gyrus (DG). Higher dose FA pretreatment markedly increased the number of Nestin- and BrdU-positive cells in the DG of postpartum female mice when compared with control mice (Figures 5C,D). No significant alterations in neurogenesis were observed in mice treated with lower doses of FA.

### Gestational Folic Acid Administration Rescued the Synaptic Mechanisms in the Hippocampus of Postpartum Female Mice

**FIGURE 6:**
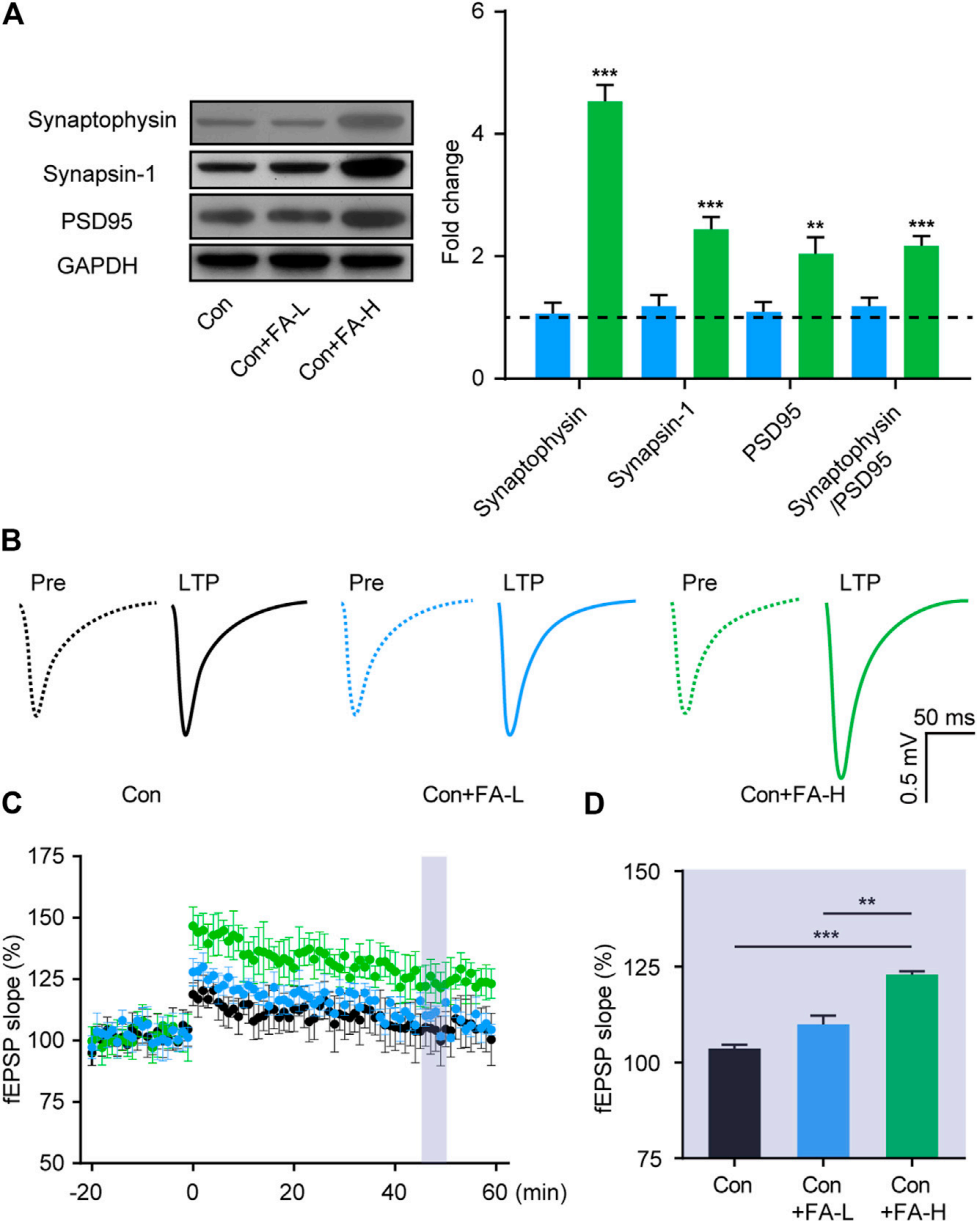
Gestational FA administration promotes synaptic plasticity in postpartum female mice. **(A)** Representative immunoblots and quantifications showing Synaptophysin, Synapsin-1 and PSD95 expression in the hippocampus. Synaptophysin One-way ANOVA: F (2, 15) = 79.19, *p* < 0.0001. Synapsin-1 One-way ANOVA: F (2, 15) = 33.79, *p* < 0.0001. PSD95 One-way ANOVA: F (2, 15) = 11.23, *p* = 0.0010. Synaptophysin/PSD95 One-way ANOVA: F (2, 15) = 17.57, *p* = 0.0001. The Con group was normalized as a dotted line, and the statistical results were compared with the Con group. **(B)** Representative traces of fEPSP responses during LTP induction. Scale, 0.5 mV, 50 ms. **(C)** Time-course responses of the evoked LTP potentials following high frequency stimulation (HFS) at CA3-CA1 synapses. The points in the time scale axis were normalized to the average baseline response to show the fEPSP slope. **(D)** Area in the shadow showed the averaged %fEPSP slope during 46–50 min after stimulation in each group. One-way ANOVA was used to analyze the LTP during 46–50 min: F (2, 15) = 13.22, *p* = 0.0005. Data were presented as mean ± SEM. *n* = 6 for each group. *p < 0.05; **p < 0.01; ***p < 0.001.

To further assess the brain mechanism implied in the therapeutic effect of FA on postpartum female mice, we examined the effects of FA on the expression of synaptic plasticity-related proteins in the hippocampus. The relative expression of synaptophysin, Synapsin-1, and PSD95 proteins was higher in the FA-H group than in the FA-L or Con mice without FA (Figure 6A). No differences were found between the FA-L and control groups without FA. We then examined basal synaptic transmission at the CA3-CA1 synapses through fEPSP recordings. In line with the previous results, the FA-H group mice showed clear synaptic enhancement. While both groups exhibited effective enhancement of synaptic transmission, LTP in FA-H slices increased to a greater extent after stimulation, compared to the control group, while no significant difference in LTP was detected between the control + FA-L and Con group mice (Figures 6B–D). These results suggest that FA administration enhances synaptic transmission in postpartum female mice.

## Discussion

The occurrence of psychiatric disorders after childbirth is well known in postpartum women. However, the treatment options for these symptoms during this special period remain limited, considering the far-reaching toxicity consequences for offspring via *in utero* or breast milk residue. A dietary FA supplements have been well recognized to be helpful, incurring beneficial effects on the nutritional requirements of lactating mothers. In the present study, we further confirmed that gestational FA administration also significantly contributed to the alleviation of emotional and cognitive dysfunction in postpartum female mice.

FA is involved in a variety of metabolic processes and represents an essential micronutrient for fetal development. Currently, there is a consensus that folate supplements during pregnancy help prevent developmental defects caused by FA deficiency in offspring. The birth of a child garners the attention of the entire family, including the parents, while the developmental defects of the offspring are easy to observe and qualitatively assess. However, the postpartum status of mothers garners much less attention and is easily ignored. Although previous studies have found that there may not be a direct correlation between low folate levels and postpartum depression (Lewis et al., 2012), epidemiological evidence has shown that supplementation of folic acid during pregnancy was inversely associated with the manifestation of pregnancy-associated depression (Xu et al., 2014; Yan et al., 2017). Moreover, associations of functional gestational FA supplements with post-partum cognition improvement have also been clinically validated (Prado et al., 2018). These results suggest that FA administration may be a suitable treatment strategy in such postpartum patients, and we tested this hypothesis for the first time in a mouse experimental model. To the best of our knowledge, this is the first experimental study to focus on the effects FA supplements on postpartum behavioral outcomes.

As a global mental health problem, depression has a higher incidence rate in women than in men. Moreover, women in the perinatal period (prepartum and postpartum) are at a higher risk of depression. Traditional antidepressants, such as serotonin reuptake inhibitors, have been found to cause negative effects on offspring when administered during pregnancy, which limits their usage and presents clinical challenges. This depressive phenotype has been well-documented in the HSP hormone withdrawal postpartum model and in postpartum animals (Li et al., 2018; Shoji and Miyakawa, 2019). Our results provide solid evidence to validate the hypothesis pointing to therapeutic effects of FA on postpartum depression (Behzadi et al., 2008). To better mimic this condition in natural situations, this FA supplementary paradigm model was applied to natural pregnancy animals during the gestational period. The effective dose in the HSP model also demonstrated an encouraging rescue effect. Although there are currently no reports in the literature regarding the effect of FA on PAD, it is striking that up to 68.9% PDD also reported anxiety symptoms (Nakić Radoš et al., 2018). Previous studies have reported that HSP mice exhibit anxiety-related behaviors (Zhang et al., 2016; Yang et al., 2017). In the present study, we showed that FA treatment greatly increased open arm exploration in the EPM and center area time in the OFT in HSP and natural postpartum mice, which indicated that FA administration also exerts anxiolytic effects. Despite the emotional symptoms, cognitive deficits have also been observed in the postpartum period (Henry and Rendell, 2007; Pio de Almeida et al., 2012) thereby demonstrating a need for new therapeutic drugs with fewer side effects. Our findings indicate that treatment with FA during pregnancy improves the hippocampus-dependent cognition score, which is consistent with previous results showing that FA could improve visuospatial ability (Prado et al., 2018). A range of emotional and cognitive symptoms were effectively resolved with regard to the treatment outcomes, considering that the hippocampus is primarily responsible for exerting emotional and cognitive functions, which prompted us to look for hippocampus-related mechanisms that could explain the efficacy of FA.

Elevated nutrition metabolism plays a crucial role in satisfying fetal metabolic demands during pregnancy, contributing to lower maternal levels of BDNF both before and after childbirth (Lommatzsch et al., 2006). BDNF is closely linked to cognition and mood, and plays an important role in neurogenesis and synaptic plasticity. To reveal the possible molecular mechanisms by which FA exerts emotional and cognitive rescue effects, we focused on the changes in BDNF-related pathway expression. In accordance with the previous finding that FA could increase hippocampal BDNF levels in stressed rats (Gao et al., 2017) and neonatal hypoxia-ischemia offspring (Deniz et al., 2018), our results showed that gestational FA caused significant changes in the expression of BDNF-related signaling in the hippocampus of postpartum female mice, thus shedding light on a potential working mechanism. Low BDNF levels correlate with low serotonin (5-HT) levels during peripartum periods (Lommatzsch et al., 2006), and BDNF could interact with the brain 5-HT-system, thus affecting 5-HT expression (Popova et al., 2017). However, the 5-HT related pathway was not assessed in this study, although this association may shed light on a deeper functional interpretation, considering that 5-HT is a key central player in the maintenance of normal brain function. Further research is warranted in order to fully understand the biological mechanisms underlying these associations.

BDNF has multiple important roles in brain development, including supporting the survival and differentiation of selected neuronal populations and modulating synaptic transmission and plasticity. Since neurogenesis plays a central role in cognition and emotion, we monitored neurogenesis after FA administration in postpartum mice. Hippocampal neurogenesis perturbations in rodents during pregnancy are well-known (Kim et al., 2010), such as in the form of PDD, PAD, and PCD, which are linked to emotional and cognitive performance. Here, we examined the immunohistochemical staining results of two stage-specific markers: nestin and BrdU. FA administration enhanced neurogenesis in the hippocampus, as indicated by an increase in Nestin- and BrdU-positive cells. The increased number of generated neurons integrate into the neural ensemble to alter behavioral phenotypes. Neuroelectrophysiological results of LTP and the expression of protein markers related to synaptic transmission consistently proved that FA has a positive impact on synaptic connectivity and subsequent cognition or emotional behavior. These hippocampal mechanisms may act symbiotically to exert their functional effects.

The regular FA dose contained in mice food chow is approximately 1–2 mg/day. Accordingly, we chose two doses of FA in this study. Based on the observed efficacy, the 5 mg/kg dose exerted a much better and more stable effect on the symptoms we observed. The potential gestional toxicity of these two FA doses was preliminary evaluated in this study. Data shown that these two doses had little effect on offspring birth and their body weight development. A dose of 5 mg/kg of FA appears to be safe in mice, both in mothers and offspring. However, additional pharmacological and toxicological studies are needed to prove safety. Though there remain limited potential health hazards data available on humans, these results offers evidence on clinical trial safety data and provide direct evidence of the preclinical potential of FA in postpartum women.

In summary, we systematically observed the effect of FA on common emotional and cognitive complications of pregnancy in experimental HSP and natural pregnant mice. The data obtained shed light on possibilities for clinical investigations of the effects of FA on brain functional parameters in postpartum women. These behavioral improvements were associated with increased BDNF-related pathway expression, neurogenesis, and synaptic transmission in the hippocampus. The validation of FA may help to unveil the biological basis of mental-related behaviors in pregnant women. Although data from further clinical trials are needed for more definitive conclusions regarding the best dietary intervention doses to improve outcomes, these results remain encouraging for pregnant women since FA may not only prevent neural tube defects, but also gestational mineral syndromes.

## Data Availability

The raw data supporting the conclusions of this article will be made available by the authors, without undue reservation.
